# Selections

**Published:** 1900-04

**Authors:** 


### SELECTIONS.

Losses of Farm Animals. The estimated percentage of mortality among farm animals during the year ending March 31, 1900, is not only below that of the year preceding, but is also below the ten and fifteen-year averages. This percentage is based upon the estimated number of farm animals on the first day of January last, and as such estimated number will be brought into conformity with those reported by the census as the result of its approaching farm-to-farm visitation, no elaborate analysis of the figures herewith presented will be made, the more important changes alone being commented upon.

*Horses.* Losses : Of the ten States each estimated to contain 500,000 horses or upward, all except Missouri report losses from disease somewhat less than those of the preceding year. The year 1898–1899, however, was characterized by a rate of mortality slightly higher than that of any previous year for which figures are available, and accordingly a comparison of the year now under discussion with the ten-year average is less satisfactory. Ohio, Indiana, Iowa, Kansas, Nebraska, and Texas report a rate of mortality below their respective ten-year averages; in the case of Pennsylvania and Missouri the percentages of loss agree exactly with the ten-year averages, while in New York and Illinois they are slightly above such averages.

Condition: The condition of healthfulness and flesh on April 1 was very high, being only 3 per cent. below the normal, as compared with 5.6 per cent. below the normal on April 1 of the preceding year. The condition by States will be found in the table on page 4, but attention should be called to the high averages reported from Pennsylvania, Illinois, Iowa, and Kansas, which were 98, 99, 100, and 99, respectively.

*Cattle.* Losses from exposure: The estimated percentage of loss from winter exposure is 1.37, against 2.2 the preceding year and a ten-year average of 1.6. This reduced mortality, as compared with the preceding year, represents a saving of nearly 400,000 cattle under this one head alone. As will be seen from the table on page 4, the highest percentages of loss are reported from the South and Southwest, where less attention is paid both to food and shelter than in the regions where more severe winter weather is expected and prepared for.

Losses from disease: The estimated percentage of loss from disease is 1.99, against 2.03 the preceding year and a ten-year average of 1.8. The figures by States, which will be found in the table on page 4, call for no special comment.

Condition: The average condition for the country at large is only 2.8 per cent. below that of normal healthfulness and flesh, as compared with 7.5 per cent. below at the corresponding date last year. The figures by States will be found on page 3, but attention may be called to the high averages, ranging from 99 to 110, from four of the New England States, from Illinois, Iowa, Kansas, Nebraska, North Dakota, Wyoming, Utah, Idaho, and the Pacific States.

*Sheep.* Losses from exposure: The estimated percentage of loss from winter exposure is 1.8, against 3.5 the preceding year and a ten-year average of 2.6. As in the case of cattle, and for the same reason, the greatest losses, relatively to the total number of sheep owned, are reported from the Southern States.

Losses from disease: The estimated percentage of loss from disease is 2, against 2.1 the preceding year and a ten-year average of 2.3. The figures by States will be found in the table on page 4, and call for no special comment.

Condition: The average condition is only 0.1 per cent. below that of normal healthfulness and average flesh, as compared with 7.6 per cent. below on April 1, 1899. This is an exceptionally high average, due mainly to the presence of a condition ranging from normal to 10 per cent. above in a few of the leading sheep States and to the entire absence of any specially low condition in any part of the country.

*Swine.* Losses from disease: No estimate of the number of swine on January 1 last having been made by the Department, the rate of mortality for the country at large cannot be determined. Of the seventeen principal States, however, twelve report a mortality below that of last year. In Texas the rate is unchanged, and only in North Carolina, Georgia, Alabama, and Arkansas is it even slightly higher than in 1898-1899. Only in five States, containing an aggregate of less than 1,000,000 head of swine, is the mortality reported above the ten-year average. The greatest losses were in the Southern States, Arkansas reporting a loss of 122 per thousand; Florida 118 per thousand, and Louisiana 101 per thousand, with a range of 39 to 94 per thousand in other parts of the South.—*United States Department of Agriculture, Division of Statistics, Crop Circular for April,* 1900.

EPICARIN. $C_6H_3$:COOH:OH.$CH_2C_{10}H_6OH$ is a condensation-product of creosotinic acid and beta-naphtol. In the ordinary form in which it is found in the market it forms a reddish-yellow, strongly acid powder, melting at 199° C., and readily soluble in alcohol and in ether. It forms easily soluble, neutral salts. According to Frick and Muller (E. Merck's *Bericht*, 1900), epicarin is but very slightly toxic, and is a good remedy for scabies in dogs, as it quickly checks the itching, and rapidly removes the affection when contracted by a human being. Though epicarin does not possess the power to kill the acarus to the same degree as does carbolic acid, creosote, tar, lysol, creolin, and iodine, its application is said to be far pleasanter. In oleaginous suspensions, or mixed with green soap, epicarin is too weak for the treatment of scabies on household animals; it should be applied in alcoholic or ethero-alcoholic solution. A solution which is recommended is the following:

| | |
|---|---|
| Epicarin . . . . . . . . . . | 10 grammes. |
| Castor oil . . . . . . . . . . | 10 " |
| Alcohol . . . . . . . . . . | 100 " |

This should be applied to the skin by means of a brush lightly drawn through the hairy coat. It is reported that these applications, should they even be licked off by the dog, are entirely free from any disadvantages, and, made every other day, rapidly effect a cure. Professor Kaposi states (*Ibid.*) that epicarin is also effective in cutaneous diseases in human beings, and that its action, resembling that of beta-naphtol, perhaps renders it of special benefit in the treatment of children. He recommends the epicarin to be used in the form of a 10 per cent. ointment with any suitable base, or in solution with alcohol and glycerin.—*Merck's Report.*

